# The Architecture of Mental Wards

**Published:** 1912-04-13

**Authors:** R. C. Wills

**Affiliations:** Manager of Inquiry Department, the Architects' Technical Bureau, 24 and 25 Hart Street, W.C.


### The Editor's Letter Box.

### THE ARCHITECTURE OF MENTAL WARDS.

Sir,—Could you kindly assist me in the following matter? One of our subscribers has written : " Can you please give us the names of some good examples of mental wards already built, connected with workhouse construction, under the Poor Law Union, governed by the L.G.B.; also, are there any books or works on same of a modern nature?" If you have any information which you could kindly place at our disposal, your help would be much appreciated.—Thanking you in anticipation, I am, yours faithfully, R. C. Wills,

Manager of Inquiry Department, the Architects' Technical Bureau,

24 and 25 Hart Street, W.C.,
April 3.

[We are not acquainted with any really first-class examples of mental wards attached to Poor Law institutions, but the majority of workhouses contain accommodation for imbeciles and epileptics, from which suggestions may be obtained. The following works should be consulted: " Suggestions and Instructions with Reference to the Construction of Lunatic Asylums, issued by the Commissioners in Lunacy" (Eyre and Spottiswoode); " Suggestions as to the Planning of Poor Law Buildings," by P. Gordon Smith (Knight and Co.); " The Planning of Poor Law Buildings," by A. C. Freeman (St. Bride's Press, Ltd.); " Asylums and Asylum Planning," by George T. Hine (Royal Institute of British Architects); " Die Kgl. Psychiatrische Klinik in München," by Kraepelin and Heilmann u. Littmann (Barth, Leipzig).—Ed., The Hospital.]

